# β-catenin/Tcf-signaling appears to establish the murine ovarian surface epithelium (OSE) and remains active in selected postnatal OSE cells

**DOI:** 10.1186/1471-213X-12-17

**Published:** 2012-06-08

**Authors:** Macalister Usongo, Riaz Farookhi

**Affiliations:** 1 Departments of Experimental Medicine, McGill University, Montreal, QC, Canada; 2 Obstetrics and Gynecology, McGill University, Montreal, QC, Canada; 3 Physiology, McGill University, Montreal, QC, Canada; 4 Department of Obstetrics and Gynecology, F344 Royal Victoria Hospital, 687 Pine Avenue West, Montreal, H3A 1A1, QC, Canada

**Keywords:** Ovarian surface epithelium, Wnts, β-catenin/Tcf-signaling, lacZ, Transgenic mice

## Abstract

**Background:**

Wnts are a family of secreted signaling molecules involved in a number of developmental processes including the establishment of cell fate, polarity and proliferation. Recent studies also implicate wnts in epithelial adult stem cell maintenance, renewal and differentiation. Wnts transduce their signal through one of three signaling pathways. The best studied, the wnt/β-catenin pathway, leads to an increase in intracellular β-catenin which acts as a co-transcription factor with members of the Tcf/Lef family. A number of wnts are expressed in the ovary, specifically in the membrana granulosa and ovarian surface epithelium (OSE). We investigated the spatio-temporal pattern of β-catenin/Tcf expression in the OSE using responsive transgenic (TopGal) mice.

**Results:**

The generated β-galactosidase response (lacZ^+^) identified the cell population that overlies the medio-lateral surface of the indifferent gonad at embryonic day (E) 11.5. From E12.5 onwards, lacZ expression disappeared in cells covering the testis but remained with ovary development. LacZ^+^ OSE cells were present throughout embryonic and postnatal ovarian development but demonstrated an age-dependent decrease to a small proportion when animals were weaned and remained at this proportion with aging. Flow cytometric (FACS) and ovarian section analyses showed lacZ^+^ cells constitute approximately 20% of OSE in postnatal (day 1) mice which fell to 8% in 5 day-old animals while in prepubertal and adult mice this accounted for only 0.2% of OSE. Apoptosis was undetected in OSE of neonates and β-catenin/Tcf-signaling cells were proliferative in neonatal mice indicating that neither cell death nor proliferation failure was responsible for the proportion alteration. It appeared that lacZ^+^ cells give rise to lacZ^-^ cells and this was confirmed in cell cultures. The DNA-binding dye DyeCycle Violet was used to set up the side population (SP) assay aimed at identifying subpopulations of OSE cells with chemoresistance phenotype associated with ABCG2 transporter activity. FACS analysis revealed lacZ^+^ cells exhibit cytoprotective mechanisms as indicated by enrichment within the SP.

**Conclusions:**

The study raises the possibility that wnt/β-catenin-signaling cells constitute a progenitor cell population and could underlie the pronounced histopathology observed for human ovarian cancer.

## Background

Wnts are secreted cysteine rich glycoprotein ligands that transduce their signal through at least three distinct pathways [1]. The wnt/β-catenin pathway (termed canonical wnt-signaling) is the best studied and arises from an increase in non-phosphorylated intracellular β-catenin content, transport to the cell nucleus and association with members of the Tcf/Lef transcription factor family to drive target gene expression [2]. Wnts are involved in a number of developmental processes including the establishment of cell fate, proliferation, and differentiation [3-5]. In mice, *Wnt4* is associated with female sexual differentiation [6] and is required during emergence of the female gonad to prevent formation of the male-specific coelomic blood vessel and steroidogenic cell migration [7,8]. In addition to *wnt4*, sex-specific expression has been found for *wnt5awnt6* and *wnt9a* within the gonad [9]. Recent findings also implicate a family of secreted ligands (R-spondin) in female sex-determination [10,11]. The R-spondins (*Rspos*) play an essential role in ovarian development through stabilization of cytoplasmic β-catenin. Mutation of *Rspo1* is associated with human sex reversal [10]. Expression of Rspo proteins overlaps with expression of *wnts* during development indicating a link between Rspo and the wnt signaling pathway [12].

While wnts play a key role in embryonic development of the ovary [6,13,14], several studies describe the expression of wnts and wnt signaling components in adult rodent ovaries [15-20]. Some of these, including *wnt4*[21] and *wnt2b*[22], are associated with activation of canonical wnt signaling. Interestingly, *wnt2b* is expressed in the ovarian surface epithelium (OSE) [18]. OSE is a simple epithelium of squamous or cuboidal cells and it, as well as Mullerian duct derivatives (oviduct, uterus, and proximal one-third of vagina), are derived from the peritoneal mesothelium [23,24]. Functionally, OSE is implicated in the ovulatory process and is responsible for repair of the ovulatory wound [25,26]. Deregulation of wnt-signaling in OSE has been implicated in ovarian tumorigenesis [27].

The spatio-temporal pattern of β-catenin/Tcf-signaling activity within murine OSE (mOSE) was investigated using a specific transgenic reporter mice (TopGal) strain [28]. We show that β-catenin/Tcf activation identifies a cell population in the mesothelium that overlies the indifferent gonad. By embryonic day (E) 12.5, the majority of cells in the overlying epithelium of the ovary retain β-catenin/Tcf-signaling cells while they disappear in cells covering the testis. At parturition the proportion of signaling to non-signaling mOSE cells decrease and fall to a small but sustained proportion in adult females. The β-catenin/Tcf-signaling population in adult females is enriched for a side population which is believed to be a characteristic of stem cells [29]. These observations might explain why ovarian cancers show oviduct/uterine histopathology as OSE share an origin with Mullerian duct derived reproductive tissues [30].

## Results

### β-catenin/tcf-activated lacZ expression is seen on cells covering the indifferent mouse gonad

**Figure 1:**
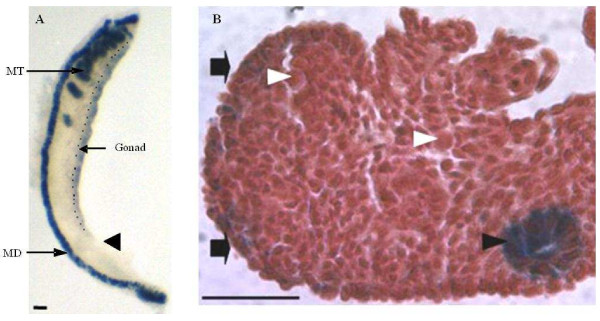
**Coelomic epithelium overlying the indifferent gonad displays ß-catenin/Tcf-mediated lacZ expression.** Panel A: Whole-mount X-gal staining of E11.5 urogenital ridge. LacZ positive cells overlie the medio-lateral surface of the indifferent gonad (dotted line demarcates the gonad). The mesonephric duct (MD) and mesonephric tubules (MT) also stain for lacZ. Arrowhead indicates coelomic epithelium extending beyond the gonad is not stained. Scale bar = 10 μm. Panel B: Section of an X-gal stained E11.5 urogenital ridge. LacZ staining is present in the gonadal surface epithelium (arrows), cells beneath the surface and the mesonephric ducts (black arrow head). Germ cells (white arrow head) do not stain for lacZ. Scale bar = 50 μm.

Prominent lacZ^+^ stained cells overlie the entire medio-lateral surface of the indifferent gonad on embryonic day 11.5 (E11.5; Figure 1A). Coelomic epithelial cells extending beyond the genital ridge towards either the rostral or caudal end of the mesonephros (Figure 1A, arrowhead), did not stain indicating that only the mesothelium directly covering the gonad has β-catenin/Tcf-mediated signaling. To identify the cell types in which β-catenin/Tcf-signaling was activated, paraffin-embedded sections of E11.5 gonads were examined. Somatic (surface and sub-surface) as well as Mullerian duct cells of E11.5 gonads showed lacZ expression (Figure 1B; dark arrows and arrow head, respectively); germ cells, distinguished by their large round nuclei, were not stained (Figure 1B, white arrowheads).

### β-catenin/tcf-mediated lacZ expression shows sexual dimorphism in the cells covering the developing gonad

**Figure 2:**
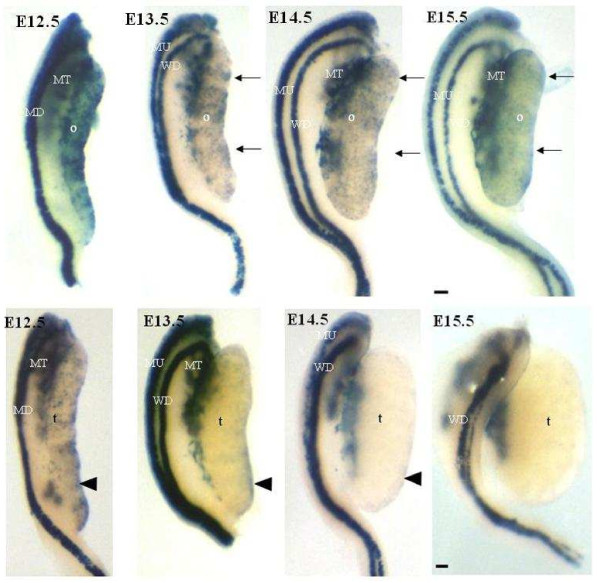
**ß-catenin/Tcf-mediated lacZ expression is maintained in the embryonic female gonad.** Time course of ß-catenin/Tcf-mediated transcription in female (upper panels) and male (lower panels) embryonic gonads. Whole-mount X-gal staining demonstrates ß-catenin/Tcf expression is sexually dimorphic from E12.5 onwards. Blue (lacZ) staining reflecting ß-catenin/Tcf-mediated transcription is observed in the mesonephric tubules (MT), mesonephric duct (MD), Mullerian duct (MU), Wolffian duct (WD), ovaries (o) and testis (t). Black arrows indicate the anterior region of the female gonad. Arrowhead indicates the ventral surface and posterior tip of the male gonad. All gonads are positioned with the anterior region at the top of each panel. Scale bar = 10 μm.

Gonadal β-catenin/Tcf-mediated lacZ-expression was examined at later stages of embryonic development. Sexual dimorphic patterns of lacZ staining were observed at E12.5 (Figure 2, upper and lower left panels). In E12.5 females, pronounced staining was observed over the entire gonadal surface while E12.5 males displayed less extensive staining compared to either the E11.5 indifferent gonad (Figure 1A) or the E12.5 ovary (Figure 2, upper left panel).

The ovarian surface at E13.5 maintained extensive lacZ expression with a bias towards the cranial pole (Figure 2, black arrows). In contrast, staining in the E13.5 testis had almost disappeared with some staining retention at the caudal pole (Figure 2, arrowhead). Staining in E14.5 ovaries was similar to that at E13.5 with an anterior bias towards the cranial suspensory ligament. E14.5 testes were devoid of lacZ^+^ cells except for the region near the mesonephric tubules and faint staining at the caudal pole. In E15.5 ovaries, lacZ staining remained prominent while testes were devoid of lacZ^+^ cells with the exception of the mesonephric tubule attachment (right panels, Figure 2). In both sexes, lacZ staining was present in Wolffian (WD) and Mullerian (MD) ducts as well as mesonephric tubules (MT). The sex-specific degeneration of the MD in the male by E15.5 (Figure 2, lower right panel) is apparent with retention of WD. The female (Figure 2 upper right panel) loses her WD at a later age, generally at E16-5 – E17.5 (not shown).

**Figure 3:**
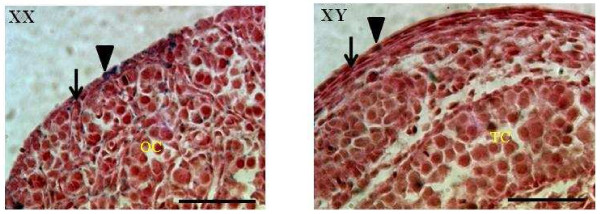
**Loss of ß-catenin/Tcf-mediated lacZ expression in the testis is not due to gonadal surface cell elimination.** E15.5 ovary (XX; left panel) and testis (XY; right panel) after Periodic acid-Schiff staining. The basement membrane (arrow) is deposited underneath the epithelial layer (arrow head) of gonads and surrounds ovigerous cords (OC) in XX and testis cords (TC) in XY gonads. Scale bar = 50 μm.

The disappearance of β-catenin/Tcf-mediated lacZ expression on the surface of developing testes raised the possibility of cell loss and coverage with a tough capsule i.e. establishment of the testicular tunica albuginea. In order to assess this possibility, basement membranes were highlighted by Periodic acid-Schiff (PAS) staining of lacZ-stained E15.5 gonad sections. A thin basement membrane (arrow; Figure 3 left panel) was observed below OSE and the lacZ^+^ and lacZ^-^ epithelial cells (arrowhead; Figure 3 left panel). Germ and pregranulosa cells, organized into structures termed ovigerous cords in developing ovaries [31], were delimited by a thin basement membrane. Gonadal surface cells of E15.5 testis did not stain for lacZ expression and appeared more elongated than E15.5 OSE cells (arrowhead; Figure 3 right panel). A basement membrane was observed underneath and between the epithelial cells of E15.5 testis and appeared to thicken at this stage (arrow; Figure 3 right panel). Testis cords consisting of germ and Sertoli cells [32] were surrounded by a basement membrane. These observations indicate that the testicular loss of β-catenin/Tcf-mediated lacZ expression is not due to surface cell elimination.

### β-catenin/tcf-mediated lacZ expression in mOSE is heterogeneous during postnatal development

**Figure 4:**
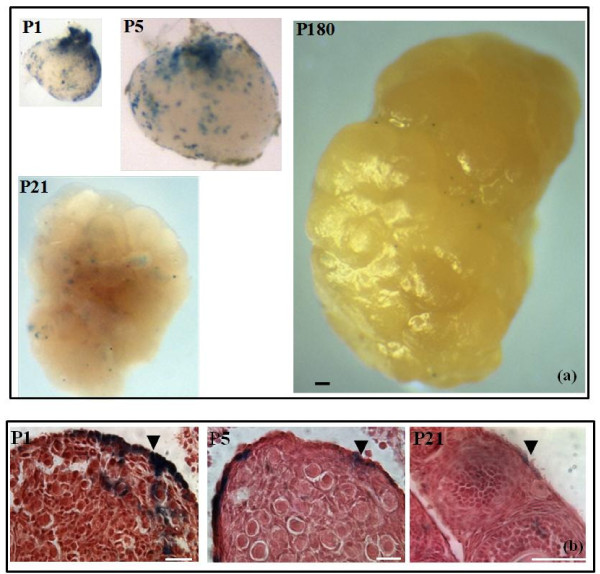
**Heterogeneity of ß-catenin/Tcf-mediated lacZ expression in the ovarian surface epithelium (OSE).** (**a**) ß-catenin/Tcf-mediated lacZ expression during ovary development from postnatal day 1 (P1) to P180 indicating an age-dependent decrease in the proportion of stained cells. Scale bar = 100 μm. (**b**) X-gal staining of OSE cells during postnatal development from P1 to P21. LacZ^+^ cells are present within the OSE (arrow heads) and indicate an age-dependent decrease in the proportion of stained OSE cells. Scale bar = 50 μm.

Whole-mount lacZ staining of ovaries derived from postnatal mice (P1–P180) revealed staining and demonstrated an age-dependent decrease in the relative proportion of stained cells (Figure 4a). Histological examination of sections derived from P1 to P21 mice showed staining in the OSE (Figure 4b) and an age-dependent decrease in lacZ^+^ OSE cells. No distribution pattern for lacZ^+^ cells within the OSE was detected at these ages.

**Figure 5:**
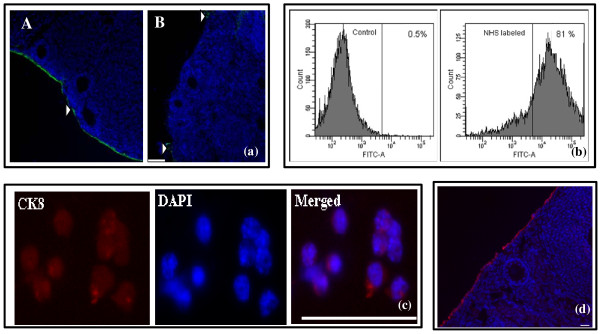
**NHS-biotin labels OSE cells.** (**a**) Panel A: A representative P21 NHS-biotin-labeled ovary section stained with avidin FITC (green) and counterstained with Hoechst 33342 (blue). NHS-biotin labels OSE cells with little penetrance of the reagent into ovarian parenchyma. Panel B: Collagenase/DNase I treatment results in isolation of a significant proportion of the OSE leaving remainder of the ovary relatively intact. Dotted line demarcates the OSE location. Arrowheads indicate labeled OSE cells. Scale bar = 100 μm. (**b**) FACS analysis of non-NHS labeled (PBS) and NHS-biotin-labeled P21 OSE cells stained with avidin-FITC. Histogram showing that the majority of the cells stained for avidin-FITC indicating that they retained the NHS-biotin label following enzymatic treatment. A minimum of 10,000 cells were analyzed. (**c**) Cytokeratin 8 (CK8) staining of OSE cells. OSE cells were isolated following enzymatic digestion of NHS-biotin labeled ovary in collagenase/DNase I in DMEM. Released cells were stained with avidin-FITC and FITC-positive cells sorted, stained for CK8 (red) and counterstained with DAPI (blue). Scale bar = 50 μm. (**d**) CK8 staining of P21 ovary. Immunostaining for CK8 labels OSE specifically (red). Scale bar = 50 μm.

In order to quantify changes in the proportion of lacZ^+^ to total OSE with animal age, cells were isolated from the ovarian surface using an enzymatic procedure and analyzed by flow cytometry. This approach was tested to ensure that OSE cells only were released. Ovary-encompassing OSE was selectively labeled using the water-soluble reagent NHS-biotin. Exposure of an intact ovary to the reagent for a brief time interval followed by quenching with excess glycine provides specific OSE labeling. Tight junctions between OSE cells and the presence of a basement membrane prevent the reagent from entering into the ovarian parenchyma [33]. Indeed, OSE cells are labeled specifically with NHS-biotin with minimal penetration into the parenchyma as visualized by the binding of FITC-conjugated avidin to NHS-biotin stained P21 ovary (Arrow head; Figure 5a panel A).

The efficiency of OSE isolation was assessed after subjecting NHS-biotin labeled ovaries to enzymatic treatment. The majority (> 90%) of the labeled cells were removed and the residual ovary remained relatively intact (Figure 5a panel B). FACS analysis of the isolated cells showed that approximately 86 ± 2.3% (mean ± sem; n = 3) of the cells were labeled with avidin-FITC (Figure 5b) after enzyme treatment. The proportion of viable cells, assessed by concurrent analysis of cell viability (7-AAD labeling), corresponded with those of unlabeled OSE from control ovaries.

After sorting NHS-biotin-labeled cells through FACS, cytokeratin 8 (CK8) detection with Cy3-labeled goat anti-mouse antibody was conducted (Figure 5c). Ovarian section from P21 mice was used as a control for the specificity of CK8 antibody to OSE cells (Figure 5d).

**Figure 6:**
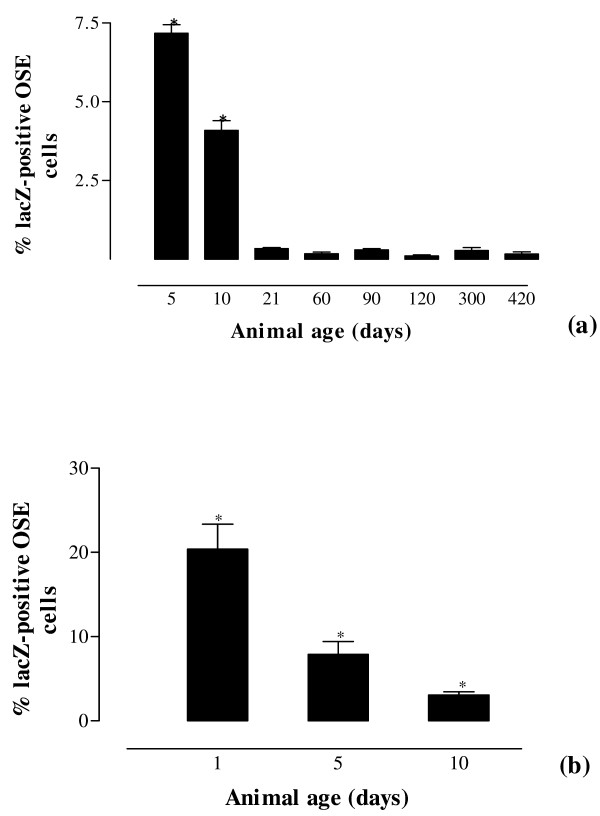
**Age-dependent decrease in ß-catenin/Tcf-signaling OSE cells.** (**a**) The percentage of lacZ-positive OSE cells as a function of animal age estimated by FACS analysis. *Error bars* represent one SD from the mean and at least three replicates were used. *, denotes *P* ≤ 0.05 between group means. A minimum of 10,000 cells were analyzed. (**b**) Serial count analysis of percentage of lacZ-positive OSE cells as a function of age. The percentage of lacZ-positive OSE cells at P5 and P10 is quite similar to that obtained at these ages using FACS. *Error bars* represent one SD from the mean and at least three ovaries were counted. *, denotes *P* ≤ 0.05 between means.

Figure 6a illustrates the percentage of lacZ-positive mOSE cells as a function of animal age. The proportion of lacZ^+^ cells within OSE decreased from approximately 8% at P5 to 0.2% by P21 and it remained at this level with continued aging (Figure 6a). FACS results were confirmed by counting lacZ-positive and negative cells in serial sections of P1, P5, and P10 ovaries (Figure 6b). The percentage of lacZ-positive OSE cells at P5 and P10 by serial counting was similar to the same measurement through FACS analysis at these ages. Sections from older animals were difficult to evaluate owing to the relative rarity of lacZ-positive OSE cells and the larger number of sections generated.

**Figure 7:**
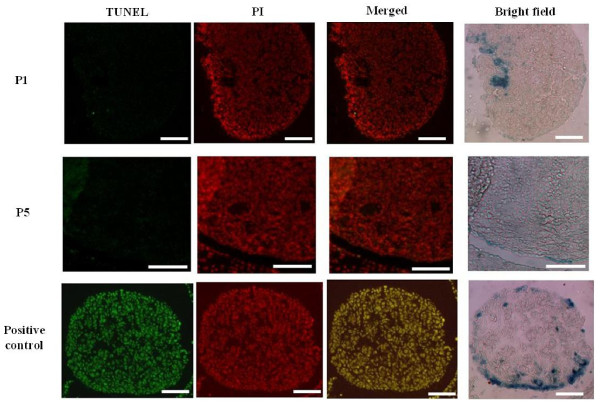
**Apoptosis is not detected in neonatal OSE cells.** Apoptosis was assessed by TUNEL on X-gal-stained P1 and P5 ovaries. TUNEL positivity (green) was not observed in the ovary. DNase-treated ovary served as a positive control. Slides were counterstained with PI (red). Scale bar = 50 μm.

Since the proportion of OSE cells expressing lacZ decreased with age, it is possible that β-catenin/Tcf expression drives cellular apoptosis. Accumulating evidence suggests that β-catenin is involved in cell cycle arrest [34] and apoptosis [35]. To investigate this possibility, the occurrence of apoptosis in P1 and P5 ovaries was analyzed by TUNEL. The positive control (DNase-treated ovary section) displayed staining in all cells (Figure 7, bottom panel). There was no evidence of DNA fragmentation in P1 and P5 ovaries (Figure 7, top and middle panel), suggesting absence of apoptosis even though significant changes in the proportion of lacZ^+^ cells were seen at these ages (Figure 6).

**Figure 8:**
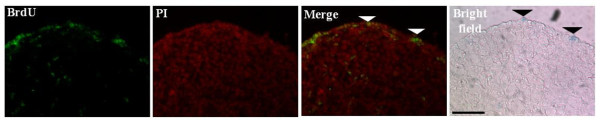
**LacZ**^**+**^**and lacZ**^**-**^**neonatal OSE cells are proliferative.** P2 mice were injected with BrdU 48 h prior to analysis. Ovaries were collected, X-gal stained, probed with anti-BrdU antibody (green) and counterstained with PI (red) to label cell nuclei. BrdU-positive cells were observed within somatic cells of the ovary (left panel) suggesting that mitosis is not restricted to the OSE. White arrow heads show BrdU^+^ and black arrow heads lacZ^+^ cells. Scale bar = 50 μm.

**Figure 9:**
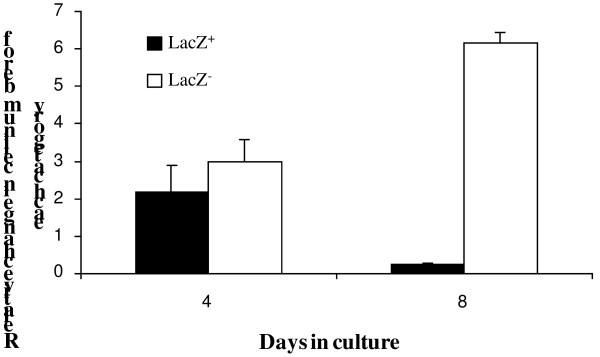
**Decrease in ß-catenin/Tcf-signaling OSE cells *in vitro.* ** OSE cells isolated from P5-P9 mice were cultured in alpha MEM supplemented with 4% FBS. The number of lacZ^+^ cells was estimated after incubation with the fluorescent substrate CMFDG. LacZ^+^ cell numbers doubled within first four days of culture and decreased subsequently with prolong culture. In contrast, lacZ-negative cells increased steadily. These results suggest that lacZ^+^ cells lose their ability to signal through the ß-catenin/Tcf-signaling pathway and become non-signaling.

It was possible that the age-dependent decrease in the proportion of lacZ^+^ cells arose because they were non-replicating. Cell proliferation was assessed immunohistochemically after incorporation of the thymidine analog, bromodeoxyuridine (BrdU), into cellular DNA. To enumerate proliferating lacZ^+^ cells, ovaries were collected 48 hr post BrdU injection and stained for lacZ and BrdU (Figure 8). At P4, approximately 25% of lacZ^+^ cells are BrdU-labeled but by P9 this falls to 8% (Table 1). This indicates lacZ^+^ cell replication decreases with age. The observation that there is an age-dependent decrease in the number and rate of replication of lacZ^+^ cells raises the possibility that proliferating lacZ^+^ cells give rise to non-signaling cells. To assess this possibility, OSE cells from P5-P9 were harvested and cultured. In four days, a 2-fold increase in lacZ^+^ cells and a three-fold increase in the number of lacZ^-^ cells were obtained (Figure 9). There was, however, no significant difference in proliferation rate between lacZ^+^ and lacZ^-^ cells. Prolonged culture for 8 days led to a significant decrease in lacZ^+^ cells whereas the number of lacZ^-^ cells increased. The latter observation suggests non-signaling cells arose from the previously signaling ones. To further confirm that lacZ^-^ arose from their positive counterparts, lacZ^+^ cells were sorted from lacZ^-^ cells and cultured separately. After 8-days of culture, the majority of lacZ^+^ cells did not stain for lacZ (data not shown). LacZ^-^ cells, however, did not stain for lacZ following prolonged culture.

**Table 1:** **Proliferating lacZ**^**+**^**cells as fraction of total lacZ**^**+**^**OSE cells. Data are presented as mean ± standard error of the mean (n = 3)**

**Animal age (days)**	**LacZ**^**+**^**BrdU/LacZ**^**+**^
4	23.6 ± 1.67
9	8.0 ± 0.48

### Wnt and fzd **expression in OSE were unchanged with age**

Table 2 is a summary of *Wnt* and *fzd* transcripts assessed for animals of different ages. Interestingly, with the exception of *fzd6* these particular *wnts* and *fzds* were either expressed or not expressed regardless of animal age. The cause for non-expression of *fzd6* in adult mice is not known nor which particular wnt signals through this receptor. *Fzd6* is an essential component of the hair patterning pathway [36] and mutation of *fzd6* is known to cause autosomal-recessive nail dysplasia [37]. Since the proportion of lacZ^+^ OSE cells in weaned (P21) and adult mice are similar, it is unlikely that the loss of *fzd6* transcription is affecting this aspect of wnt-signaling in OSE cells.

**Table 2:** Expression of wnts and frizzleds in OSE cells harvested from different animal ages and primer sequences for RT-PCR analysis

**Gene**	**P5**	**P10**	**P21**	**Adult (>6 wks old)**	**Forward primer**	**Reverse primer**
Wnt2	+	+	+	+	CGACTGGGGTGGCTGCAGTG	AGGGAGCCTGCCTCTCGGTC
Wnt2b	+	+	+	+	CATGATCAACGAGGGGACTT	CAGCCTTGTCCAAGACACAGT
Wnt3a	–	–	–	–	CAAGCCGGCGATGGCTCCTC	ACTCCCGGGTGGCTTTGTCCA
Wnt4	+	+	+	+	GGCGTAGCCTTCTCACAGTC	GCCGTCAATGGCTTTAGATG
Wnt5a	+	+	+	+	AGGAGTTCGTGGACGCTAGA	GTCCATCCCCTCTGAGGTCT
Wnt7a	–	–	–	–	CTGGCGGGATCAGCACAGCC	CAGCCTCCCGACTCCCCACT
Wnt8a	–	–	–	–	TCGTGGACAGTTTGGAGAAA	GTTGCGGTTGCAGTAGTCAG
Wnt11	+	+	+	+	CGTGTGCTATGGCATCAAGT	CGTGTGCTATGGCATCAAGT
GAPDH	+	+	+	+	ACAACTTTGGCATTGTGGAA	GATGCAGGGATGATGTTCTG
Fzd 1	+	+	+	+	CCGAGCTCAAGTTCTTCCTG	GGGAACTTCTCGCACTTGAG
Fzd 2	+	+	+	+	TTCTTCACGGTCACCACCTA	AATGTAGGCCACTGACACCA
Fzd 3	+	+	+	+	CCAGGAACCTGACTTTGCTC	GACACTCCCTGCTTTGCTTC
Fzd 4	+	+	+	+	TGCAGTTCTTCCTTTGTTCG	TCTCAGGACTGGTTCACAGC
Fzd 5	+	+	+	+	GACGCCGAGGTTCTGTGTAT	TCGTTCCATGTCAATGAGGA
Fzd 6	+	+	+	+	CACAAATCATGGCACCTCTG	GGTTGGTTCTGGAGAACTGG
Fzd 7	+	+	+	+	GCCTACAACCAGACCATCCT	GCACACGGGTGCGTACATAG
Fzd 8	–	–	–	–	CCTTCGCCACTGTCTCTACC	ACGTGAGCGACAGGATTACC
Fzd 9	+	+	+	+	CCAGTACGTGGAGAAGAGTCG	GAAGGTGAACACGGTGAAGG
Fzd 10	+	+	+	+	GAGCACGGGCTGTACCTTAG	ATGAAGGAAGTGCCGATGAC

+/– indicates presences or absences of RT-PCR product.

### LacZ^+^ OSE cells are enriched with a potential stem cell population

**Figure 10:**
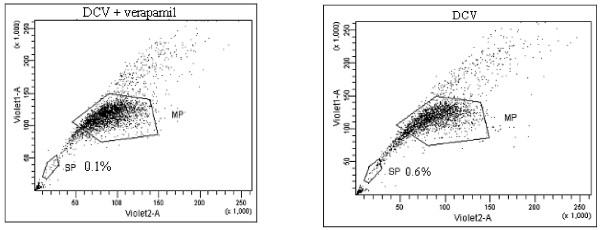
**OSE cells show a side population (SP).** Dye cycle violet (DCV) SP analysis of OSE cells. OSE cells harvested from prepubertal mice were stained with DCV in the presence or absence of the ABCG2 inhibitor verapamil. Addition of verapamil resulted in reduction in SP. The ratio of SP cells to total viable cells is indicated as a percentage in the scatter plot. Boxed cells (labeled MP) represent the main population.

**Figure 11:**
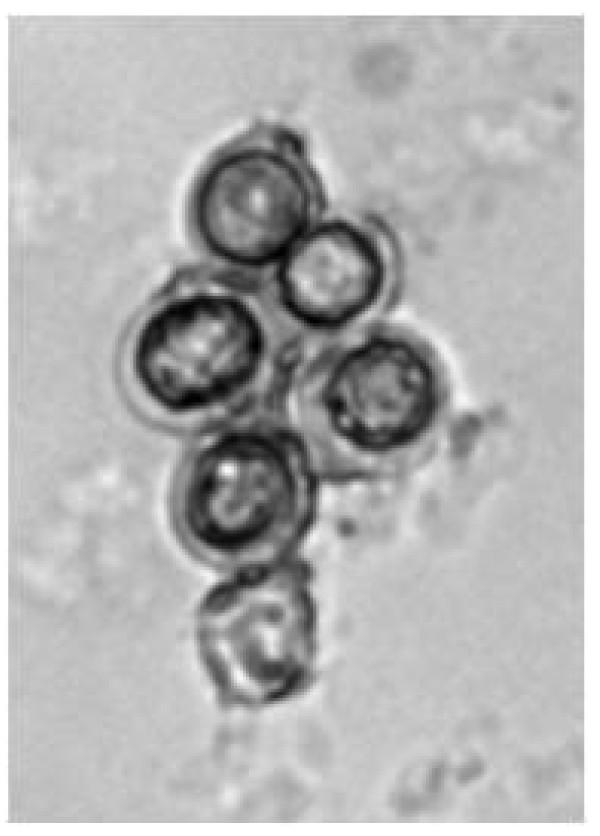
SP cells from mouse OSE. Isolated OSE cells were stained with DCV and SP cells sorted into 3% paraformaldehyde.

The observation that there is an age-dependent decrease to relative constancy of β-catenin/Tcf-signaling OSE cells prompted an examination of whether these cells exhibit a stem cell characteristic termed side population (SP). SP is detected by dual wavelength flow cytometry of the cellular efflux of the DNA-binding dyes Hoechst 33342 or dyecycle violet (DCV). SP cells appeared as a characteristic tail in the flow cytometry of isolated OSE (Figure 10). The SP cells were small round cells of approximately six μm in diameter (Figure 11). The dependence of the SP phenotype on expression of ABCG2 transporter pump was demonstrated by pre-incubation with the ABCG2 inhibitor verapamil. The results indicate that while ~ 0.6% OSE of prepubertal mice is SP (Table 3), ~ 23.9 ± 2% (mean ± SE, n = 3) of lacZ-positive cells are SP positive. This also indicates that the lacZ^+^ cell population is heterogeneous with potential stem and non-stem cell components.

**Table 3:** **Percentage of side population (SP) cells relative to total viable OSE cells and β-catenin/Tcf-signaling cells as a fraction of total lacZ**^**+**^**OSE cells in SP and main population (MP)**

**Animal age (days)**	**% SP/total viable cells**	**% lacZ**^**+**^**cells in SP**	**% lacZ**^**+**^**cells in MP**
24	0.8	25	75
26	0.6	20	80
35	0.5	27	73

## Discussion

Transgenic reporter (TopGal) mice indicate activation of β-catenin/Tcf signaling within OSE. This dominates the epithelium covering the indifferent gonad and is followed by its maintenance in a sex-specific manner. The majority, most likely all of the cells overlying the differentiating ovary, retain β-catenin/Tcf activity, which begins to disappear with testis formation. Further ovarian development shows an age-dependent decrease in the proportion of β-catenin/Tcf-responsive cells to relative constancy in the mature ovary. RT-PCR analysis detected the expression of multiple wnts and fzds within the OSE. Side population analysis indicates enrichment in β-catenin/Tcf-signaling cells. These findings suggest that β-catenin/Tcf signaling cells form the definitive OSE and raise the possibility that these signaling cells constitute a putative stem cell population.

β-catenin/Tcf-signaling plays critical role in embryonic patterning and cell fate determination in a variety of tissues [38]. Wilms’ tumor suppressor 1 [39] and empty-spiracles homeobox gene 2 [40] expressed within the thickening coelomic epithelium of the developing gonad are required for genital ridge development. The presence of a lacZ^+^ cell population overlying the E11.5 gonad suggests that the mouse gonad may be formed from a β-catenin/Tcf-signaling cell population. Coelomic epithelial cells migrate into XX and XY gonad and contribute to supporting cell lineage of the gonad [32]. The localization of lacZ^+^ cells within E11.5 gonads raises the possibility that β-catenin/Tcf-mediated expression may be involved in differentiation of somatic cells of the developing gonad.

β-catenin is dispensable for testis formation and maintenance whereas it is required for maintenance of ovarian characteristics [41]. Several genes known to play a role in sexual development are transcribed in a sexually dimorphic fashion [42,43]. We observed sexual dimorphic β-catenin/Tcf-mediated expression that is distinct by E12.5. It is established that the presence of germ cells in female gonads is required for proper development of the ovary [44] and that sexual differentiation and meiotic entry of germ cells in embryonic XX gonads progress in an anterior-to-posterior pattern [45]. Our analysis demonstrated that lacZ expression in ovaries show the opposite wave pattern: an anterior bias observed by E13.5 and extends to the early postnatal period. This staining pattern wave is similar in its spatial distribution to that of *Adamts19* a marker of ovarian somatic differentiation [45,46] and raises the possibility that OSE differentiation begins posteriorly and moves anteriorly. This pattern of β-catenin/Tcf-mediated expression in the XX gonad may be due to a gradient of meiosis promoting substances produced by a fixed source in or near the anterior portion of the ovary. There is some evidence that the ovary in germ cell deficient mice is covered by OSE cells [47]. Thus, the observed ovarian lacZ expression pattern is unlikely due to a substance produced by germ cells but could be reflective of local environmental cues that are present in the embryonic female gonad.

*Sry* expression blocks β-catenin-mediated transcription [48]. The observation that loss of lacZ expression begins at the anterior portion of the developing testis mimicking *Sry* expression suggests that the anterior-posterior loss of lacZ on the surface of embryonic testis may be due to *Sry* expression. Because *Sry* is not expressed in the coelomic epithelium of a differentiating testis [49], it is unlikely that the loss of lacZ is due to *Sry* expression. Alternatively, non-cell autonomous paracrine signals such as *Sox9Mis*, and *Dhh* emanating from Sertoli cells [50] may be responsible for blocking β-catenin/Tcf expression. It is possible that active steroidogenesis present in embryonic testis but absent in fetal ovary [51] may be preventing the β-catenin/Tcf-pathway from being efficiently expressed in the developing testis.

Several methods have been described for isolating OSE cells [52-55]. We have established a simple procedure for isolating a relatively pure population of OSE cells. This procedure owes its success to the fact that the NHS-biotin label is retained following enzymatic digestion. Additionally, biotinylation did not affect OSE cell viability. Sorted NHS-labeled cells stained for CK8 indicating that they were derived from the OSE.

Some studies support a positive role of β-catenin in cell mitosis [56], whereas others suggest a potential involvement in cell cycle arrest [34] or a direct involvement in apoptosis [35]. β-catenin accumulation within the cell nucleus is however involved in cell fate decision [57]. We have shown that age-dependent decrease in β-catenin/Tcf-signaling cells is not due to the absence of cell proliferation or the result of selective apoptosis. Similar studies in day-2 and day-4 neonatal mouse ovaries reported the absence of apoptosis in all ovarian cell types [58]. We suspect that lacZ^+^ cells were quiescent with continued aging and prepared for differentiation. It is likely that upon mitosis, a lacZ^+^ cell looses its ability to signal through the β-catenin/Tcf pathway and becomes non-signaling. This was reflected by the loss in proliferative capacity of lacZ^+^ cells from P4 to P9. *In vitro*, lacZ^+^ cells showed a transient increase in cell number followed by appearance of lacZ^-^ cells suggesting that loss of lacZ expression is due to cellular differentiation. The transient increase in lacZ^+^ cells may originate from symmetric division of lacZ^+^ cells or insufficient downregulation of β-catenin/Tcf-lacZ expression *in vitro* following mitosis. The observation that sorted lacZ^+^ cells cultured separately from their non-signaling counterparts gave rise to lacZ^-^ cells provides the supporting evidence that loss of lacZ expression is due to OSE differentiation.

Knockout studies of various wnt molecules led to the discovery that wnts perform critical functions during early development of reproductive tissues [6,59]. Our studies show expression of various wnt ligands and fzd receptors transcripts within OSE. These are similar to previous studies assessing wnt/fzd expression within the ovary [15,19]. Among the wnts found to be expressed in the OSE are *wnt-4* and *wnt-2*, which have been shown to impact ovary development [6,60]. The overlapping expression of multiple *wnts* within the OSE is suggestive of functional redundancy.

Gene expression analysis supports the hypothesis that human OSE cells are multipotent [61]. This hypothesis has recently been validated by a study that described putative stem cells within the OSE as cells expressing markers of pluripotency [62]. A side population-enriched and label-retaining cell population in the coelomic epithelium of adult mouse ovary has been identified as possible stem/progenitor cells [26]. We obtained a distinguishable SP within mouse OSE. The observation that the SP is enriched for β-catenin/Tcf-signaling cells raises the possibility that lacZ-positive cells constitute OSE progenitors. The fact that cancer stem cells are a subset defined by increased wnt/β-catenin activity [63] and that wnt/β-catenin is essential for maintenance of intestinal stem cells [64] supports our hypothesis. There is accumulating evidence suggesting that somatic stem cells may undergo mutagenic transformation into cancer stem cells [65]. Because many of the properties that define somatic stem cells also define cancer stem cells, our identification of a β-catenin/Tcf-signaling cell population in OSE raises the possibility that endometrioid adenocarcinomas may arise as a result of transformation of the lacZ^+^ cells. The common origin of the β-catenin/Tcf-signaling cells with oviduct/uterus may explain the suggestion that OSE cancers show uterine/oviductal characteristics [30].

## Conclusion

Our findings indicate that β-catenin/Tcf-signaling cells are present early in OSE development. The maintenance of a constant number of β-catenin/Tcf-signaling cells accompanied by the increase in appearance of non-signaling cells raises the possibility that the original β-catenin/Tcf-signaling cells give rise to a replacement as well as an expanding population of non-signaling progeny. Taken together, our results indicate that the mouse OSE is heterogeneous and may contain a population of progenitor cells. The physiological necessity for restoring OSE after ovulations may reflect the need for establishing and maintaining the progenitor cells. It also raises the possibility, specifically in primates, that transformation of OSE progenitor cells can generate the varied histopathology observed in ovarian cancer.

## Methods

### Reagents and chemicals

Deoxyribonuclease I, collagenase (Type IV), and 5-bromo-2′-deoxyuridine (Brdu) were purchased from Sigma Aldrich (St Louis, MO). Glycine, N, N-dimethyl formamide, and other general chemicals were of tissue culture grade and purchased from Fisher Scientific (Nepean, ON). Dulbecco’s Modified Eagle Medium (DMEM), Minimum Essential Medium (MEM) alpha, 5-bromo-4-chloro-3-indolyl-β-D-galactopyranoside (X-gal), dithiothreitol (DTT), propidium iodide (PI), 4′, 6-diamidino-2-phenylindole (DAPI), 7-aminoactinomycin D (7-AAD), and 5-chloromethylfluorescein di-b-D-galactopyranoside (CMFDG) kit were purchased from Invitrogen (Burlington, ON). Bovine serum albumin (BSA) and fetal bovine serum (FBS) were purchased from Wisent (St-Bruno, QC), Hoechst 33342 was purchased from Roche (Laval, QC), Tween-20 from Bio Basic Inc (Markham, ON), mowiol from Calbiochem (La Jolla, CA), and N–Hydroxysulfosuccinimide (Sulfo-NHS)-biotin from Pierce Thermo Fisher (Nepean, ON). Strepavidin phycoerythrin-Cy5 (PE-Cy5) was obtained from Biolegend (San Diego, CA). The mouse monoclonal antibodies against cytokeratin-8 (TROMA-1) and BrdU (G3G4) were obtained from Developmental Studies Hybridoma Bank (DSHB; Iowa City, IA), Cy3-labeled goat anti-mouse IgG antibody (Jackson Immuno Research, West Grove, PA) and avidin-FITC (EY Laboratories, San Mateo, CA) were obtained through Cedarlane Laboratories (Burlington, ON). The In Situ Cell Death Detection Kit, Fluorescein was purchased from Roche (Laval, QC)

### Animals

All animal procedures followed the guidelines established by the Canadian Council of Animal Care and approved by the Animal Care Committee of the Royal Victoria Hospital, McGill University. CD1 mice bearing the β-catenin/Tcf-responsive lacZ reporter gene (TopGal mice) have been described [28]. Dr. Daniel Dufort (Department of Obstetrics and Gynecology, McGill University, Montreal, Canada) provided us with female and male CD1 mice, homozygous for the transgene, for colony establishment. Dr Makoto C. Nagano (Department of Obstetrics and Gynecology, McGill University, Montreal, Canada) provided us with wild type CD1 mice. Female mice were examined daily for vaginal plugs. The day of plug detection was considered day 0.5 of gestation and the day after birth designated postnatal day 1 (P1). Gonads were isolated from a minimum of three mice for analysis and all experiments replicated at least thrice.

### Tissue processing

Mice were sacrificed by cervical dislocation. Gonads were isolated, washed in phosphate buffered saline (PBS; 137 mM NaCl, 2.7 mM KCl, 8 mM Na_2_HPO_4,_ 2 mM KH_2_PO_4,_ pH 7.4) and fixed for 5–15 min in freshly prepared 4% paraformaldehyde (PFA) in PBS. After rinsing in wash buffer (PBS containing 2 mM MgCl_2,_ 0.1% Triton, 0.05% sodium deoxycholate), gonads were stained in the dark overnight at 37°C in wash buffer supplemented with 1 mg/ml X-gal, 0.04% N, N-dimethyl formamide, 5 mM potassium ferricyanide and 5 mM potassium ferrocyanide to disclose β-galactosidase activity [66]. Following staining, gonads were washed in PBS and photographed or processed for histology.

### Histology

X-gal-stained ovaries were post-fixed in 4% PFA overnight at room temperature (RT), rinsed in PBS, and embedded in paraffin. Sections were cut 6 μm thick, mounted on glass slides and counterstained with hematoxylin and eosin. In studies where OSE cell numbers were estimated, sections were stained with periodic acid Schiff (PAS) to define the basement membrane and counterstained with Hoechst 33342 to identify cell nuclei. The total number of OSE cells was estimated by applying the nucleator and fractionator principle described by Gundersen [67]. Only cells with large visible nuclei were counted. Every fourth ovary section was evaluated and an estimate of the total number of OSE cells per ovary determined by multiplying the cell counts by four. LacZ-positive OSE cells were determined by evaluating every section of the ovary since these cells were not uniformly distributed.

### OSE labeling and isolation

OSE cells were isolated as follows: ovaries (2 ovaries/0.5 ml DMEM) were placed in a 1.5 ml capped tube and incubated for 60 min at 37°C in DMEM containing 1 mg/ml Type IV collagenase, 1 mg/ml deoxyribonuclease I, and 0.53 mM EDTA. Ovaries were agitated every 10 min by swirling the tube for a few seconds. Released cells were transferred to a fresh tube. The ovaries were rinsed in fresh DMEM and additional released cells combined with the previously isolated cell suspension. The cell suspension was vortexed and cells pelleted by centrifugation at 500 g for 5 min. The cell pellet was washed with PBS and resuspended in PBS.

We needed to confirm that our isolation procedure yielded primarily OSE cells with minimal contamination by other ovarian cells. Additionally, we wanted to assess the efficiency of the OSE isolation procedure. This was accomplished by labeling OSE cells *in situ*. We took advantage of a water-soluble and membrane impermeable biotinylation reagent (Sulfo NHS-biotin) that reacts chemically with exposed amine groups of cell surface proteins. Intact ovaries were incubated in 1 mg/ml Sulfo NHS-biotin in PBS for 1 min at 4°C. The reaction was quenched by incubating the ovary in ice-cold PBS containing 0.1 M glycine for 1 min. Selective labeling of OSE cells was confirmed by preparing sections of NHS-biotin-labeled ovaries followed by incubation with avidin-conjugated FITC (1:200 in PBS). Sections of enzymatically-treated ovaries were also examined for the extent of OSE removed by avidin-FITC staining.

### Immunofluorescence

Cytokeratin 8 (CK8) staining was performed on paraffin-embedded sections and isolated OSE cells. Six μm thick paraffin tissue sections were deparaffinized with xylene and rehydrated in graded ethanol. Antigen retrieval was performed by boiling the sections in 10 mM sodium citrate buffer, pH 6.0, for 25 min. After rinsing in PBST (PBS + 0.5% Tween), nonspecific binding was blocked for 30 min in blocking solution (5% BSA in PBST). Sections were incubated with primary antibody (TROMA-1) in blocking solution at 4°C overnight. The primary antibody was omitted for negative control slides. Slides were subsequently rinsed in PBST, incubated with Cy3-labeled goat anti-mouse antibody diluted 1:200 in blocking solution for 60 min in the dark, counterstained with DAPI, and mounted in mowiol.

Isolated OSE cells (50,000 cells/ml) were cytospun onto slides, fixed in 4% PFA for 5 min, and processed for CK8 staining as described above. Cell counts were made in five microscopic fields and approximately 100 cells were counted per field.

### Fluorescence-activated cell sorting (FACS) analysis

LacZ expression in isolated OSE cells was detected using the DetectaGene Green CMFDG LacZ gene expression kit as outlined by the supplier (Invitrogen). OSE cells were washed in PBS and incubated with pre-warmed 0.1 M CMFDG in PBS at 37°C for 15 min. PI was added to label dead cells and FACS analysis performed on a Becton-Dickinson FACScan. Wild type CD1 OSE cells were used as controls. Dual parametric analysis of forward versus side scatter was the primary gate for identification of cells in the appropriate size range and to eliminate cell debris.

### TUNEL (terminal deoxynucleotidyl transferase dUTP nick end labeling) staining

Six μm thick paraffin-embedded lacZ-stained ovary sections from each age under investigation were examined for the presence of fragmented DNA indicating apoptosis using the In Situ Cell Death Detection Kit, Fluorescein, according to the manufacturer’s instructions (Roche). Sections were counterstained with PI to label cell nuclei and mounted in mowiol.

### BrdU labeling

A solution of 1 mg 5′-bromo-2′-deoxyuridine (BrdU)/100 μl PBS was freshly prepared and filter sterilized (0.45 μm filter). Mice received BrdU by injection (i.p.) of 100 μl of sterile preparation. Ovaries were collected 48 hrs later and stained for X-gal, post fixed in 4% PFA overnight and embedded in paraffin. To detect proliferating cells, 6 μm thick paraffin sections were analyzed using a monoclonal mouse antibody (DSHB) specific for BrdU. Sections were dewaxed, washed in PBST (PBS + 0.5% Tween 20) and incubated with 2 N HCl at RT for 1 hr. Subsequently, slides were rinsed 3x5 mins in PBST and blocked in a blocking solution (3% BSA in PBST) for 30 mins. Sections were then incubated with anti-BrdU antibody diluted 1:100 in blocking solution at 4°C overnight. Following primary antibody incubation, slides were rinsed 3×5 mins in PBST, incubated with an FITC-labeled goat anti-mouse secondary antibody diluted 1:100 in blocking solution overnight at 4°C, counterstained with DAPI, and mounted in mowiol.

### RT-PCR

Total RNA was isolated from FACS-sorted OSE cells using the miRNeasy Mini kit (Qiagen, Toronto, ON) and incorporating on-column RNase-free DNase digestion. Quantity and quality of mRNA samples were assured by analysis with the Thermo Scientific NanoDrop 2000 (Thermo Scientific, Wilmington, DE). To survey the expression of wnt signaling components, total RNA (100 ng) was reverse transcribed using M-MLV reverse transcriptase (Invitrogen). Polymerase chain reactions (PCRs) were conducted as described previously [68]. Wnts for RT-PCR analysis were selected based on previous studies [15,19]. A PCR reaction for a known housekeeping gene, GAPDH, was generated as an internal control. The annealing temperature for each primer pair was optimized using positive control tissues to generate single bands corresponding to correct product sizes. Information regarding positive control tissue in which the genes of interest are expressed was obtained from: (i) Mouse Genome Database (MGD) (http://www.informatics.jax.org 09/2009), RT-PCR database and included brain (for wnt2, wnt 4, wnt 7a, wnt 8a, wnt 11), eye (wnt5a), testis (wnt3a); and (ii) a previous study [69]. The RT-PCR protocol was performed on two separate RNA preparations. For each sample, a RT-minus control was included to provide for a negative control for subsequent PCR. All minus RT controls were negative. PCR products were visualized on a UV transilluminator after electrophoresis on a 1% agarose gel in TAE buffer (40 mM Tris, 1 mM EDTA and 20 mM acetic acid) and SafeView nucleic acid staining.

### Side population (SP) analysis

OSE cells were labeled with DyeCycle Violet (DCV) according to a modified protocol [70]. Briefly, OSE cells were suspended in the appropriate medium (DMEM containing 2% FBS and 2 mM HEPES) at 1 x 10^6^ cells/ml. Before DCV incubation, cells were pre-incubated for 15 mins with or without 50 *μ*M verapamil. DCV was added to the cell suspension at a final concentration of 5 *μ*M and the mixture incubated for 30 minutes at 37°C in the dark. Propidium iodide was added to a final concentration of 1 *μ*g/mL to identify dead cells. FACS analysis and sorting were performed on a dual laser flow cytometer (Becton Dickinson). The SP was defined as described previously [71].

### Cell culture

OSE cells isolated from postnatal day 5 (P5) to P9 mice were grown in MEM alpha [72] supplemented with 4% FBS and 20 U/ml PenStrep at a density of ~3000 cells/well in 96-well plates. The cells were grown in a humidified incubator at 37°C and 5% CO_2_.

### Statistical analysis

Data were analyzed using SYSTAT 10.2 statistical software (SYSTAT Software, Richmond, CA). Analysis of variance (ANOVA) was used with Tukey’s test in the post hoc analysis for cell counts to ascertain group mean differences. Data for the percentage of lacZ-positive OSE cells obtained following CMFDG labeling was subjected to ANOVA after arcsine transformation [73]. Data are presented as mean ± standard error of the mean. P ≤ 0.05 was considered significant.

## Authors’ contributions

RF designed research; UM performed research and analyzed data; RF and UM wrote the paper. Both authors read and approved the final manuscript.
